# Acute Effect of Topical Menthol on Chronic Pain in Slaughterhouse Workers with Carpal Tunnel Syndrome: Triple-Blind, Randomized Placebo-Controlled Trial

**DOI:** 10.1155/2014/310913

**Published:** 2014-09-15

**Authors:** Emil Sundstrup, Markus D. Jakobsen, Mikkel Brandt, Kenneth Jay, Juan Carlos Colado, Yuling Wang, Lars L. Andersen

**Affiliations:** ^1^National Research Centre for the Working Environment, Lersø Parkalle 105, 2100 Copenhagen O, Denmark; ^2^Institute for Sports Science and Clinical Biomechanics, University of Southern Denmark, 5230 Odense M, Denmark; ^3^Laboratory of Physical Activity and Health, Research Group in Sport and Health, Department of Physical Education and Sports, University of Valencia, 46010 Valencia, Spain; ^4^Department of Rehabilitation Medicine, The Sixth Affiliated Hospital of Sun Yat-sen University, No. 26 Yuancun 2nd Cross Road, Guangzhou 510655, China

## Abstract

Topical menthol gels are classified “topical analgesics” and are claimed to relieve minor aches and pains of the musculoskeletal system. In this study we investigate the acute effect of topical menthol on carpal tunnel syndrome (CTS). We screened 645 slaughterhouse workers and recruited 10 participants with CTS and chronic pain of the arm/hand who were randomly distributed into two groups to receive topical menthol (Biofreeze) or placebo (gel with a menthol scent) during the working day and 48 hours later the other treatment (crossover design). Participants rated arm/hand pain intensity during the last hour of work (scale 0–10) immediately before 1, 2, and 3 hours after application. Furthermore, global rating of change (GROC) in arm/hand pain was assessed 3 hours after application. Compared with placebo, pain intensity and GROC improved more following application of topical menthol (*P* = 0.026 and *P* = 0.044, resp.). Pain intensity of the arm/hand decreased by −1.2 (CI 95%: −1.7 to −0.6) following topical menthol compared with placebo, corresponding to a moderate effect size of 0.63. In conclusion, topical menthol acutely reduces pain intensity during the working day in slaughterhouse workers with CTS and should be considered as an effective nonsystemic alternative to regular analgesics in the workplace management of chronic and neuropathic pain.

## 1. Introduction

Carpal tunnel syndrome (CTS) is a neuromuscular condition caused by increased pressure on the median nerve at the level of the wrist and accounts for approximately 90% of all entrapment neuropathies [1, 2]. Commonly reported symptoms of CTS include pain in the wrist and hand [3], paresthesias [4], thenar muscle weakness, and loss of dexterity [4]. Female gender, increasing age, physical illness, repetitive hand use, and occupation are potential risk factors for the development of CTS [1, 5]. However, Falkiner and Myers [6] concluded that except in the case of work that involves very cold temperatures (possibly in conjunction with load and repetition) such as butchery work is less likely than demographics and disease related variables to cause CTS. In line with this, the prevalence of CTS among Danish slaughterhouse workers was found to be almost 4 times that of reference workers (6.3% versus 1.6%) possibly due to the highly repetitive and forceful work tasks [7].

Conservative treatment is usually offered to individuals with mild to moderate intermittent symptoms of CTS, whereas surgical carpal tunnel release is the preferred treatment of patients with persistent CTS symptoms and those not responding to conservative treatment [2, 4, 8, 9]. Oral medications such as nonsteroidal anti-inflammatory drugs (NSAIDs) and corticosteroids along with corticosteroid injection are offered as a nonsurgical treatment of CTS. These local analgesics have been shown to relieve neuropathic pain by acting as a sodium channel blocker in the affected nerves; however high systemic concentrations of these compounds may increase the likelihood of adverse events [10]. For example, gastrointestinal problems and dyspepsia have been reported following use of NSAIDS [11] and corticosteroid use can lead to adverse osseous and ocular effects [12]. Thus, alternative treatments for temporal pain relieve such as topical analgesics that act at the peripherally located site of injury could provide the symptomatic benefits of oral analgesics on neuropathic pain but without the risk of adverse events [13–15].

Menthol possesses weak analgesic properties when applied to the site of musculoskeletal injury and topical gel containing menthol is thus used as analgesics [16–18]. Topical menthol application produces a cool sensation by activation of the TRPM8 channel also known as the cold and menthol receptor 1, found mainly within thermosensitive neurons [19, 20]. Menthol increases the sensitization of these neurons consequently leading to the perception of coolness, which have an inhibitory effect on nociceptive afferents and on dorsal-horn neurons conducting pain impulses to the thalamus [21]. It is well established that specific sodium channels (Nav 1.8 and Nav 1.9) are greatly involved in pain pathways and tissue specific localization, and the development of type-specific blockers of sodium channels is an important part of the treatment of chronic and neuropathic pain [22, 23]. Hence, Gaudioso et al. [24] found menthol to be a state-selective blocker of Nav 1.8, Nav 1.9, and TTX-sensitive sodium channels in rats and highlighted the role of menthol as topical analgesic compound by its ability to be a sodium channel inactivator.

Johar and coworkers [25] demonstrated that a menthol based topical analgesic was more effective than ice for decreasing DOMS induced symptoms of pain in the elbow flexors and Higashi et al. [15] reported, in a double-blind randomized controlled trial, significant pain relief from muscle strain following 8-hour application of a patch containing methyl salicylate and menthol compared to placebo. Additionally, a randomized controlled study found that topical menthol combined with chiropractic adjustments reduced acute low back pain [26]. Thus, application of topical menthol is used to relieve pain of the musculoskeletal system and is widespread in sport medicine; however, double-blind randomized placebo-controlled trials are lacking and the acute effects of topical menthol application on chronic neuropathic pain remain unclear.

The aim of the study was to evaluate the acute effect of topical menthol and placebo (gel with a menthol scent) on pain in slaughterhouse workers with chronic pain and symptoms of carpal tunnel syndrome.

## 2. Methods

### 2.1. Study Design

This triple-blind randomized placebo-controlled crossover trial evaluates the acute effect of topical menthol (Biofreeze) and placebo (gel with a menthol scent) on chronic pain in Danish slaughterhouse workers with symptoms of carpal tunnel syndrome. The study was approved by The Danish National Ethics Committee on Biomedical Research (Ethical Committee of Frederiksberg and Copenhagen; H-3-2010-062) and registered in ClinicalTrails.gov (NCT01716767). The Consolidated Standard of Reporting Trials (CONSORT) checklist was followed to ensure transparent and standardized reporting of the trial. All participants were informed about the purpose and content of the project and gave their written informed consent to participate in the study. All experimental conditions conformed to The Declaration of Helsinki.

### 2.2. Recruitment and Flow of Participants

Recruitment was established on subjects excluded from participation in another randomized controlled trial [27] due to contraindications of carpal tunnel syndrome. In that study, 19 individuals showed symptoms of carpal tunnel syndrome and were subsequently excluded from an intervention with high-intensity resistance training and were invited to participate in this study. Of the 19 invited individuals, 10 met the inclusion criteria and were willing to participate in the project.

The recruitment was as follows. A screening questionnaire was administered to 645 Danish slaughterhouse workers (aged 18–67 years). In total 595 individuals replied to the questionnaire of which 410 were interested to participate in the research project. The initial inclusion criteria based on the screening questionnaire were (1) currently working at a slaughterhouse for at least 30 hours a week, (2) pain intensity in the shoulder, elbow/forearm, or hand/wrist of 3 or more on a 0–10 VAS scale during the last 3 months, (3) stating at least “*some*” work disability scoring on a five-point scale: “*not at all,*”* “a little,” “some,” “much,*” to “*very much*” when asked the following question: “*during the last 3 months, did you have any difficulty performing your work due to pain in the shoulder, arm, or hand,*” (4) no participation in resistance training during the last year, and (5) no ergonomics instruction during the last year. Of the 410 interested respondents, 145 met the above inclusion criteria and were invited for a clinical examination.

A total of 135 employees presented for the baseline clinical examination. Exclusion criteria were hypertension (Systolic BP > 160, diastolic BP > 100), a medical history of cardiovascular diseases, symptoms of carpal tunnel syndrome, recent traumatic injury of the neck, shoulder, arm, or hand regions, or pregnancy. Furthermore, at the day of the clinical examination participants filled in another questionnaire with the following inclusion criteria: (1) pain intensity in the shoulder, elbow/forearm, or hand/wrist of at least 3 on a 0–10 VAS scale during the last week, (2) pain that lasted more than 3 months, and (3) frequency of pain of at least 3 days per week during the last week.

**Figure 1:**
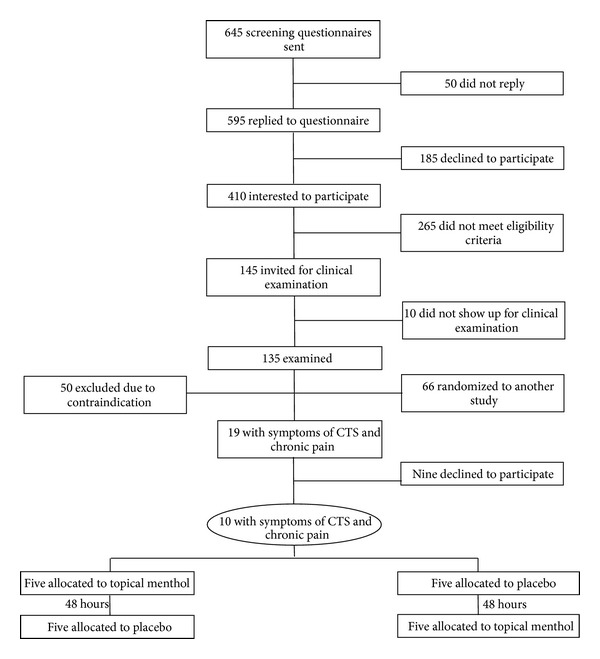
Participants flow. CTS denotes carpal tunnel syndrome.

Based on the clinical examination and associated questionnaire, 69 workers were excluded due to contraindications of which 19 showed symptoms of carpal tunnel syndrome. Symptoms of carpal tunnel syndrome included (1) nocturnal numbness of the hand; (2) paresthesia in the distribution of the median nerve; (3) positive Tinel's sign over the carpal tunnel; (4) positive Phalen's test; (5) decreased sensibility in the distribution of median nerve; (6) decreased strength in abduction of the thumb; (7) pain intensity of at least 4 in the hand/wrist; and (8) the pain should have lasted at least 3 months. Participants should fulfill all these eight criteria to be defined as having carpal tunnel syndrome. The 19 workers with symptoms of carpal tunnel syndrome were invited to participate in the present study and 10 workers willingly accepted.  Figure 1 shows the flow of participants through the study.

### 2.3. Randomization and Blinding

Using a computer generated random numbers table, participants were randomly distributed into two groups to receive either topical menthol (Biofreeze) or placebo on the first day of testing at a 1 : 1 menthol/placebo ratio. Interspersed by a minimum of 48 hours, participants received the other treatment (crossover design) on the second day of testing.

Both the active treatment (topical menthol gel) and placebo (gel with a menthol scent) were provided by The Hygenic Corporation (Akron, OH).

Menthol and placebo gels were prepared by technicians from The Hygenic Corporation who also verified proper labeling of the gel tubes with corresponding allocation code. The menthol and placebo gels were packaged and labeled in the same manner, so that each topical gel tube resembled the other. The menthol and placebo topical gels, which had no other identifiers, were delivered to a blinded study administrator at the National Research Centre for the Working Environment and further delivered by hand to the research assistant who administered the treatment and recorded the allocation on a separate case report form. Following data collection and statistical analyses the allocation code was broken by The Hygenic Corporation and delivered to the researchers at the National Research Centre for the Working Environment.

### 2.4. Interventions

Participants were invited for two separate days of testing involving topical application of menthol or placebo to the arm, wrist, and hand. On the first day of testing, participants were randomly allocated to receive either topical menthol (Biofreeze) or placebo (gel with a menthol scent) and on the second day of testing, participants received the contrasting treatment, thus acting as their own controls in a crossover design.

The Biofreeze topical gel contained 4% active menthol and the following ingredients:* Aloe barbadensis *leaf extract,* Arnica Montana* flower extract,* Arctium lappa* root extract,* Boswellia carterii *resin extract,* Calendula officinalis* extract, carbomer,* Camellia sinensis* leaf extract, camphor, glycerin,* Ilex paraguariensis* leaf extract, isopropyl alcohol, isopropyl myristate,* Melissa officinalis* leaf extract, silicon dioxide, tocopheryl acetate, triethanolamine, Blue 1, and Yellow 5. The placebo comparator contained no menthol but had a menthol scent, with the following ingredients:* Aloe barbadensis* leaf extract,* Arnica montana* flower extract,* Arctium lappa* root extract,* Boswellia carterii* resin extract,* Calendula officinalis* extract, carbomer,* Camellia sinensis* leaf extract, camphor, fragrance, glycerin,* Ilex paraguariensis* leaf extract, isopropyl alcohol, isopropyl myristate,* Melissa officinalis* leaf extract, silicon dioxide, tocopheryl acetate, triethanolamine, Blue 1, and Yellow 5. The menthol and placebo gels had a similar texture, odor, and color.

Menthol and placebo were applied topically to the hand and wrist by a blinded research assistant at a recommended dosage of 2.5 mL per 500 cm^2^ [28]. As the majority of the slaughterhouse workers with CTS also suffered from general elbow and forearm pain, topical menthol and placebo were additionally applied to the forearm. The mode of application involved light strokes with no substantially force, pressure, or rubbing [25]. Topical application was applied to the participants at the end of lunch break during a typical working day at the slaughterhouse, allowing for a 3-hour testing period after lunch. Additionally, all participants were asked about adverse events during every reporting of pain intensity (at 1, 2, and 3 hours following application) by the blinded research assistant.

### 2.5. Outcome Measures

The primary outcome was the change in arm/hand pain intensity (scale 0–10) during work. The participant rated “pain intensity during the last hour” on the 0–10 modified VAS scale (where 0 indicates “no pain at all” and 10 indicates “worst pain imaginable”) immediately before and 1, 2, and 3 hours after application of the gel [29, 30]. The primary outcome is calculated as the change in pain from before to after (average of 1, 2, and 3 hours after) application of the gel. The secondary outcome measure was the global rating of change (GROC), which is a fundamental clinical tool to elucidate whether a patient has improved or worsened and is commonly used among patients with musculoskeletal symptoms, including chronic pain [31, 32]. Thus, the scale is used to asses participants overall evaluation of the topical gel application treatment [33]. Participants rated the change in arm/hand pain on a scale from −5 (much worsening of pain) to 5 (much improvement of pain) 3 hours after application of the gel.

### 2.6. Sample Size

Power calculations showed that 10 participants in a paired crossover design were necessary for testing the null hypothesis of equality of treatment at an alpha level of 5%, a statistical power of 80%, a minimal relevant difference in hand/wrist pain intensity of 1.5, and SD of 1.5 on a scale of 0–10.

### 2.7. Statistical Analysis

Statistical analyses were performed using the SAS statistically software for Windows (SAS Institute, Cary, NC). The primary outcome (change in hand/wrist pain) was analyzed according to intention-to-treat principle using a repeated measures 2 × 2 mixed-factorial design (Proc Mixed), with* time*,* group,* and* time by group* as independent categorical variables (fixed factors). Subject was entered as a repeated effect. Analyses were adjusted for gender and pain intensity at baseline. The secondary outcome variable (GROC) was analyzed by analysis of variance (ANOVA) and adjusted for gender and pain intensity at baseline. Prior to the ANOVA, a Kolmogorov-Smirnov goodness-of-fit test had shown that the data did not significantly deviate from a normal distribution.

An alpha level of 0.05 was used for statistical significance. The primary outcome variable (change in hand/wrist pain) is reported as between-group least square mean differences and 95% confidence intervals from before to after (average of 1, 2, and 3 hours after) application of the gel. The secondary outcome variable is reported as between-group least square mean differences and 95% confidence intervals. Finally we calculated effect size as Cohen's d [34] based on arm/hand pain intensity (between-group differences divided by the pooled standard deviation).

## 3. Results

Table 1 shows baseline characteristics of the participants. All participants completed the intervention and none of the participants reported any adverse events to either placebo or menthol topical application.

### 3.1. Pain and Global Rating of Change

**Figure 2:**
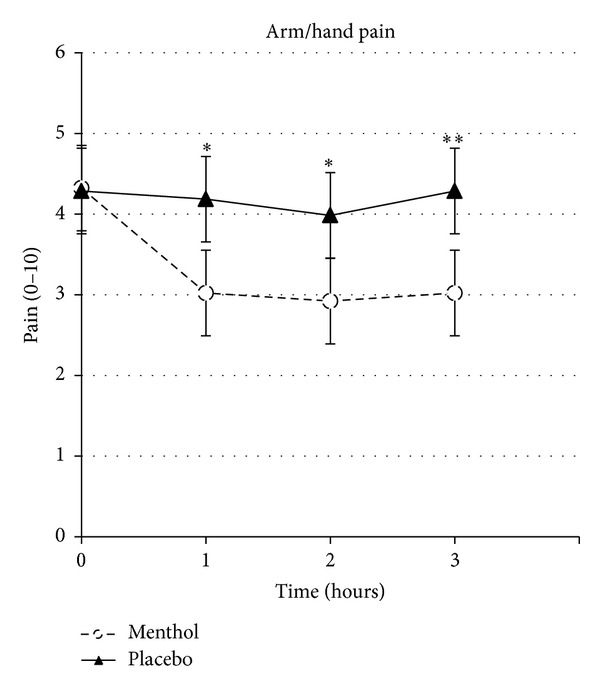
Change in arm/hand pain following application of topical menthol (menthol) or topical placebo (placebo) before (time 0) and 1, 2, and 3 hours following application. *, ** denotes significant difference between interventions (*P* < 0.05; *P* < 0.01, resp.).

Figure 2 illustrates the change in hand/wrist pain immediately before and 1, 2, and 3 hours after application of Biofreeze and placebo, respectively. A priory hypothesis testing showed a statistically significant* group by time* interaction for pain intensity (*P* = 0.026) from before to after (average of 1, 2, and 3 hours after) topical application. Compared with placebo, hand/wrist pain intensity decreased −1.2 (CI 95%: −1.7 to −0.6) following Biofreeze application. The effect size (Cohen's d) of the change in arm/hand pain was 0.63 and categorized as moderate with topical menthol. Post hoc analyses revealed a significant pain intensity reduction at all timepoints (1, 2, and 3 hours) following menthol application compared to placebo (*P* = 0.016, *P* = 0.027, and *P* = 0.009, resp., Table 2).

Analysis of variance showed a* group* effect for GROC of hand/wrist pain 3 hours following topical application (*P* = 0.044). Compared to placebo, GROC improved to a greater extent with Biofreeze (1.5 point; CI 95%: −2.94 to −0.1, Table 2).

## 4. Discussion

This triple-blind, randomized placebo-controlled trial found that topical gel containing menthol applied to the hand and arm acutely reduced chronic pain among slaughterhouse workers with carpal tunnel syndrome. The effect persisted all three hours of the experiment.

Topical gel containing menthol led to a 31% (1.3 point on 0–10 VAS) acute reduction in chronic pain associated with carpal tunnel syndrome, and the absolute change in pain symptoms between topical menthol and placebo was 1.2 corresponding to a moderate effect size (Cohen's d ≥ 0.50). In patients with chronic pain a change in pain intensity of 1 on a 0–10 scale is considered a minimal clinical important change [35], and Todd et al. [36] reported the minimum clinically significant change in patients with acute pain measured with a 100 mm scale to be 13 (corresponding to a 1.3 change on a 0–10 scale). Thus, the results of the present study indicate a marginal clinical significant change in chronic pain perception following menthol based application. This is further supported by the observed difference in global rating of change (GROC) in hand/wrist pain in favor of the topical menthol group. Topical menthol application could therefore serve as an alternative to oral analgesics, to produce clinical relevant reductions in chronic pain intensity without resulting in high systemic concentrations of analgesic that may lead to adverse events.

Menthol based topical applications are a widely used analgesic compound acting at the peripherally located site of injury. Zhang et al. [26] reported a significant reduction in acute low back pain following 4 weeks of Biofreeze application combined with chiropractic adjustment compared with chiropractic adjustment alone. However, the study was not blinded, and the acute effects of topical menthol application were not measured. Another study found topically applied menthol to cause higher transepidermal water loss (which is often compromised due to injury) whereas no effect on pain sensation was observed, compared to alcohol [18]. Topical menthol is regularly used in sports medicine, and a topical patch of methyl salicylate—an analgesic—in combination with menthol has been reported to relief pain associated with mild to moderate muscle strain compared to placebo [15]. However, the direct contribution of menthol on pain is difficult to extract as the patch also contained methyl salicylate.

Menthol applied to the skin increases the sensitization of thermosensitive neurons by activation of the TRPM8 channel consequently leading to the perception of coolness, which has an inhibitory effect on nociceptive afferents and on dorsal-horn neurons conducting pain impulses to the thalamus [21]. It has been reported that the subjective cooling effect following topical menthol application lasts up to 70 min in 12 of 18 subjects, with a mean cooling sensation of 32 min [18]. This knowledge contributed to the study design by Johar et al. [25] who measured DOMS induced pain symptoms and tetanic contraction force 20, 25, and 35 min following application of either menthol gel or ice to the elbow flexors. They demonstrated that the menthol containing gel was more effective than ice for increasing evoked tetanic force; however no significant group by time interaction in pain perception was observed. Our study revealed a decrease in pain intensity 1 hour following menthol application, and this reduction was maintained for both 2 and 3 hours indicating that pain relief lasts longer than the perceived cooling effect. This discrepancy between the sensation of cool and the perception of pain should in a timewise perspective be investigated in future studies.

Workplace risk factors for the development of CTS involve repetitive and forceful hand use, and the prevalence of CTS among Danish slaughterhouse workers was found to be almost 4 times that of reference workers [6, 7]. Workers diagnosed with carpal tunnel syndrome may be treated by surgical procedures, while others will have to rely on conservative treatments. However, physical exercise such as strength training, which has shown to relieve other types of musculoskeletal pain [37, 38] (refs), may be contraindicated in carpal tunnel syndrome. Thus, topical menthol may provide acute pain reduction for this group of workers. For instance, the gel can be applied in the morning and again at lunch to provide pain relief during the entire working day. Nevertheless, it should be remembered that the acute analgesic effect of topical menthol does not treat the underlying cause of carpal tunnel syndrome, and the workloads may need to be adjusted to prevent further aggravation of the symptoms.

### 4.1. Strengths and Limitations

The randomized, triple-blind placebo-controlled crossover design protects against systematic bias. As we did not measure nerve conduction velocity or ultrasound waves over the carpal tunnel we were not able to conclusively establish the diagnose of carpal tunnel syndrome. However, to be regarded as a worker with symptoms of carpal tunnel syndrome all participants were to experience all of the following symptoms: (1) nocturnal numbness of the hand; (2) paresthesia in the distribution of the median nerve; (3) positive Tinel's sign over the carpal tunnel; (4) positive Phalen's test; (5) decreased sensibility in the distribution of median nerve; (6) decreased strength in abduction of the thumb; (7) pain intensity of at least 4 in the hand/wrist; and (8) pain that lasted at least 3 months. A limitation of the study is that only subjective rating scales are used as outcome variables. However, even without objective measures to support the subjective variables, the triple-blind, placebo-controlled design eliminates the probability of a placebo effect. The exclusion and inclusion criteria used in the present study confine the generalizability of our results to workers with chronic pain and symptoms of carpal tunnel syndrome exposed to highly and repetitive and forceful work. The size of the study allows us to test the effectiveness of topical menthol, but for evaluating adverse events a much larger study is needed. However, topical gels are generally considered safe.

## 5. Conclusion

Topical menthol application acutely reduces pain intensity among slaughterhouse workers with chronic pain and symptoms of carpal tunnel syndrome compared with placebo. Thus, topical menthol should be considered as an effective nonsystemic alternative to regular analgesics in the workplace management of chronic, localized musculoskeletal, and neuropathic pain.

## Figures and Tables

**Table 1:** Baseline characteristics of the participants. Values are means (SD).

Demographics	
Height, cm	173 (7)
Weight, kg	80 (21)
Body mass index, kg m^−2^	26 (5)
Age, year	45 (7)
Number of men/women	8/2
Clinical	
Elbow/forearm pain intensity during the last week (scale 0–10)	6.3 (2.3)
Hand/wrist pain intensity during the last week (scale 0–10)	5.7 (2.8)
Arm/hand pain intensity during the last hour of work (scale 0–10)	4.3 (1.8)
Days with pain during the last week	5.8 (1.9)

**Table 2:** Changes in arm/hand pain intensity and global rating of change (GROC) following menthol and placebo topical application. Differences of each group are shown on the left and post hoc contrasts between the groups on the right. Values are means (95% confidence interval).

	Within-group difference from before to after application		Between-group difference	
	Menthol	Placebo	Menthol versus placebo	*P* value
Pain at hour 1 (0–10)	−1.3 (−2.2 to −0.4)	−0.1 (−1.0 to 0.8)	−1.2 (−2.1 to −0.2)	0.016
Pain at hour 2 (0–10)	−1.4 (−2.3 to −0.5)	−0.3 (−1.2 to 0.6)	−1.1 (−2.0 to −0.1)	0.027
Pain at hour 3 (0–10)	−1.3 (−2.2 to −0.4)	0.0 (−0.9 to 0.9)	−1.3 (−2.2 to −0.3)	0.009
GROC (−5 to 5)	−1.2 (−2.4 to −0.1)	0.3 (−0.9 to 1.4)	−1.5 (−2.9 to −0.1)	0.044
